# Clinical course and therapeutic approach to varicella zoster virus infection in children with rheumatic autoimmune diseases under immunosuppression

**DOI:** 10.1186/s12969-016-0095-3

**Published:** 2016-06-02

**Authors:** Raphael Leuvenink, Florence Aeschlimann, Walter Baer, Gerald Berthet, Elvira Cannizzaro, Michael Hofer, Daniela Kaiser, Silke Schroeder, Ulrich Heininger, Andreas Woerner

**Affiliations:** University of Basel Medical School, Basel, Switzerland; University Children’s Hospital, Zürich, Switzerland; Department of Pediatrics, Hospital of Chur, Chur, Switzerland; Department of Pediatrics, Hospital of Aarau, Aarau, Switzerland; Unité romande de rhumatologie pédiatrique, CHUV, University of Lausanne and HUG, Geneva, Switzerland; Department of Pediatrics, Hospital of Lucerne, Lucerne, Switzerland; Pediatric Rheumatology, University of Basel Children’s Hospital, Spitalstrasse 33, CH – 4031 Basel, Switzerland

**Keywords:** Varicella zoster virus, Pediatric, Rheumatic autoimmune disease, Immunosuppression

## Abstract

**Background:**

To analyze the clinical presentation and complications of varicella zoster virus (VZV) infection in children with rheumatic diseases treated with immunosuppressive medication such as biological disease-modifying antirheumatic drugs (bDMARDs) and/or conventional disease-modifying antirheumatic drugs (cDMARDs), and to analyze the therapeutic approach to VZV infections with respect to the concomitant immunosuppressive treatment.

**Methods:**

Retrospective multicenter study using the Swiss Pediatric Rheumatology registry. Children with rheumatic diseases followed in a Swiss center for pediatric rheumatology and treated with cDMARD and/or bDMARD with a clinical diagnosis of varicella or herpes zoster between January 2004 and December 2013 were included.

**Results:**

Twenty-two patients were identified, of whom 20 were treated for juvenile idiopathic arthritis, 1 for a polyglandular autoimmune syndrome type III, and 1 for uveitis. Of these 22 patients, 16 had varicella and 6 had herpes zoster. Median age at VZV disease was 7.6 years (range 2 to 17 years), with 6.3 years (range 2 to 17 years) for those with varicella and 11.6 years (range 5 to 16 years) for those with herpes zoster. The median interval between start of immunosuppression and VZV disease was 14.1 months (range 1 to 63 months). Two patients had received varicella vaccine (1 dose each) prior to start of immunosuppression. Concomitant immunosuppressive therapy was methotrexate (MTX) monotherapy (n = 9) or bDMARD monotherapy (n = 2), or a combination of bDMARD with prednisone, MTX or Leflunomide (n = 11). Four patients experienced VZV related complications: cellulitis in 1 patient treated with MTX, and cellulitis, sepsis and cerebellitis in 3 patients treated with biological agents and MTX combination therapy. Six children were admitted to hospital (range of duration: 4 to 9 days) and 12 were treated with valaciclovir or aciclovir.

**Conclusion:**

The clinical course of varicella and herpes zoster in children under immunosuppression is variable, with 4 (18 %) of 22 children showing a complicated course. Thorough assessment of VZV disease and vaccination history and correct VZV vaccination according to national guidelines at diagnosis of a rheumatic autoimmune disease is essential to minimize VZV complications during a later immunosuppressive treatment.

## Background

In the absence of a universal childhood immunization program for varicella zoster virus (VZV), VZV infection is widely spread in the Swiss pediatric population. By the age of 14 years, 95 % of children are seropositive for VZV [1]. The clinical features of VZV infection are determined by primary infection leading to varicella (chickenpox) and endogenous reactivation leading to herpes zoster (shingles). The most frequent complications of varicella are secondary infections, notably cellulitis, abscesses, pneumonia, sepsis and fasciitis, mainly caused by *Staphylococcus aureus* or group A β-hemolytic streptococci. Complications of the central nervous system may present as cerebellar ataxia, meningoencephalitis or cerebral vasculitis [2].

Children diagnosed with a rheumatic autoimmune disease (e.g., juvenile idiopathic arthritis) are frequently treated with immunosuppressants to reduce disease activity. Immunosuppressive medication includes conventional disease-modifying anti-rheumatic drugs (cDMARDs), such as methotrexate or leflunomide, and a growing number of biological disease-modifying anti-rheumatic drugs (bDMARDs), such as inhibitors of tumor necrosis factor-alpha (e.g., etanercept, adalimumab, infliximab), T-cell co-stimulation (abatacept), interleukin-1 (anakinra, canakinumab), interleukin-6 (tocilizumab) or inductors of B-cell depletion (rituximab). Each of these drugs are known to increase susceptibility to infectious diseases and complications thereof [3].

The aim of this retrospective study was to assess the clinical characteristics of VZV disease in children with rheumatic diseases under immunosuppression. A second aim was to assess the therapeutic approach to VZV disease under immunosuppression.

## Methods

### Study design

This was a retrospective multicenter study based on the Swiss Pediatric Rheumatology Register. The Swiss Pediatric Rheumatology Register was established in 2004, including all patients seen in the 8 centers for pediatric rheumatology in Switzerland (Aarau, Basel, Bern, Chur, Lausanne, Lucerne, St. Gallen and Zurich). Available data from the register were date of birth, sex, age at diagnosis, diagnosis and disease details. All centers but one agreed to participate in this study. Detailed information addressing different clinical and therapeutic parameters of VZV disease were obtained by a specific questionnaire (available from the authors by request).

### Patients

Patients who developed varicella or herpes zoster during the study period (January 2004 to December 2013) while being treated with conventional and/or biological DMARDs in one of the participating Swiss centers for pediatric rheumatology were included for analysis.

### Data collection

The seven participating centers received a standardized questionnaire and were asked to identify patients who fulfilled the inclusion criteria. The collected detailed data were returned to the investigators in Basel (RL, UH, AW). Besides inclusion of the basic characteristics from the register, the questionnaire included the following variables: age at occurrence of VZV disease, VZV vaccination status, previous varicella or herpes zoster, medication at onset of VZV disease, rash (varicella: atypical, i.e., <100 vesicles or typical, i.e., ≥100 vesicles; herpes zoster: segment), complications, hospitalization, treatment. Furthermore it included impact on immunosuppressive medication and possible changes in rheumatic disease activity as assessed by the treating physician six months after the occurrence of VZV manifestation.

### Data analysis

Data from the questionnaire was entered into a database (Microsoft Office Excel, Microsoft, USA). Descriptive analysis was performed with SPSS, IBM, USA.

### Ethics

The Swiss Pediatric Rheumatology Register received ethical approval by the University of Lausanne (No. 101/02).

## Results

On 31st December 2013, 2452 patients with rheumatic diseases were included in the registry.

Of these, twenty-two patients fulfilled the inclusion criteria, with 16 patients presenting with varicella and 6 patients with herpes zoster. The median age of the patients was 7.6 years (range 2 – 17 years). Sixteen were female. Two patients had been vaccinated (1 dose) against VZV. A serology proving seroprotection against VZV after vaccination had not been performed. Twenty patients had a diagnosis of juvenile idiopathic arthritis; one patient was diagnosed with polyglandular autoimmune syndrome type III and one patient with anterior uveitis. Immunosuppressive therapy at the onset of VZV manifestation was methotrexate (MTX) monotherapy (n = 9) or bDMARD monotherapy (etanercept, n = 2), or a combination of bDMARD with prednisone, MTX or Leflunomide (n = 11).

### Patients with varicella

Baseline characteristics for patients are shown Table 1. Of the 16 patients with varicella, 11 were female and the median age of the patients was 6.3 years (range 2.5 – 17.4). Nine of 16 varicella manifestations in total occurred within the first 12 months after the introduction of immunosuppressive therapy (median 10.5 months, range 1.1 – 30.3 months). Four of 16 varicella manifestations occurred with a mild presentation (less than 100 vesicles) while the other 12 cases showed a typical manifestation (more than 100 vesicles). Two patients had previously received a single-dose of varicella vaccine. Both showed a non-complicated clinical course, but yet one of them received intravenous aciclovir for 7 days to treat the infection.

Complications of VZV manifestation emerged in 4 of 16 varicella patients. The complications included cellulitis in three patients (one additionally presented with a septic condition) and cerebellitis in one patient. Five of 16 patients were admitted to hospital for a median of 6 days (range 4 –9 days) (Table 1). Ten patients, including the 4 with complications, had been treated with antiviral medication: 9 patients had received aciclovir, one patient valaciclovir. Median duration of treatment was 7 days (range 5 to 8 days).

**Table 1:** Baseline characteristics, VZV disease characteristics and impact on immunosuppressive treatment

Baseline characteristics						VZV disease characteristics						Impact on immunosuppressive treatment
Case	Sex/Age (years)	Diagnosis	Medication at onset of VZV	VZV vaccine doses	Time to event (months)	Date of onset	Rash	Complications	Hosp.	Treatment	Dur.	Management of immunosuppression
*Varicella*												
1	F/4.8	JIA	MTX	0	15.1	05/2004	typical	cellulitis	6d	ACV i.v. + AB	7d	unknown
2	F/6.3	JIA	MTX	0	14.9	06/2005	typical	none	no	no	-	continued
3	F/6.3	JIA	MTX	0	14.2	01/2006	typical	none	no	no	-	continued
4	F/3.0	JIA	MTX	0	6.2	01/2007	typical	none	no	no	-	continued
5	F/6.1	JIA	MTX + INF	0	17.6	02/2008	typical	cellulitis, sepsis	9d	ACV i.v. + AB	7d	interrupted
6	F/3.7	JIA	ETA	0	4.2	02/2009	typical	none	no	ACV i.v.	n.a.	interrupted
7	F/3.4	JIA	MTX + ETA	0	8.6	04/2009	typical	none	no	no	-	interrupted
8	F/2.5	JIA	ETA	0	4.4	03/2010	typical	cellulitis	no	ACV p.o. + AB	7d	continued
9	F/17.4	JIA	MTX	1	29.9	10/2010	typical	none	no	no	-	continued
10	M/8.9	JIA	MTX + ADA	0	21.7	02/2011	typical	cerebellitis	5d	ACV i.v.	7d	continued
11	M/8.8	JIA	MTX + INF	0^a^	5.7	04/2011	atypical	none	no	no	-	continued
12	F/7.2	JIA	MTX	0	3	02/2012	typical	none	5d	ACV i.v.	5d	interrupted
13	F/6.2	JIA	MTX + TCZ	0	1.1	03/2012	atypical	none	no	ACV p.o.	8d	interrupted
14	M/4.0	JIA	MTX	0	12.4	04/2012	atypical	none	no	VCV p.o.	7d	interrupted
15	M/4.1	JIA	TCZ + Pred.	0	3.2	06/2012	typical	none	6d	ACV i.v.	6d	interrupted
16	M/7.5	JIA	MTX + INF	1	30.3	04/2013	atypical	none	no	ACV i.v. + p.o.	7d	interrupted
*Herpes zoster*												
17	F/6.4	JIA	MTX	0	5.5	04/2004	n.a.	none	no	no	-	continued
18	F/14.7	JIA	MTX + ADA	0	10.9	10/2004	n.a.	none	no	no	-	continued
19	F/5.9	JIA	MTX	0	4.1	01/2005	n.a.	none	no	no	-	continued
20	M/13.6	PAS III	RTX + Pred.	0	6	12/2008	n.a.	none	no	no	-	continued
21	F/9.5	JIA	LEF + INF	0	27.6	07/2011	Lumbar 1, left	none	no	VCV p.o.	n.a.	interrupted
22	F/16.5	Ant. Uv.	MTX + INF	0	63	11/2013	Trigeminal ophthalmic nerve, left	none	4d	ACV iv. + p.o.^b^	7d	interrupted

^a^had a history of varicella at the age of 6 months. *Hosp* hospitalization, *Dur* duration, *MTX* methotrexate, *ETA* etanercept, *INF* infliximab, *ADA* adalimumab, *TCZ* tocilizumab, *Pred* prednisone, *VCV* valaciclovir, *ACV* aciclovir, *AB* antibiotics; ^b^also received local aciclovir. *LEF* leflunomide, *RTX* rituximab, *JIA* juvenile idiopathic arthritis, *Ant. uv* anterior uveitis, *PAS* polyglandular autoimmune syndrome, *n/a* not available

### Patients with herpes zoster

Six patients developed herpes zoster while under immunosuppression for underlying rheumatic disease; their median age was 11.6 years (range 5.9 – 16.5). Median time to event was 8.4 months (range 4.1 – 63 months). Four of the six cases with herpes zoster occurred within the first 12 months of the onset of the immunosuppressive therapy. 

No complications were observed in the six patients with herpes zoster; one patient was admitted to hospital (Table 1). Two patients had been treated with antiviral medication (aciclovir and valaciclovir, one each). In patients 21 and 22, the dermatomes were reported, with the former suffering from herpes zoster in the lumbar dermatome L1 on the left hand side and the latter being affected in the dermatome of the first branch of the trigeminal nerve.

### Impact on immunosuppressive treatment and change in activity of baseline disease

Immunosuppressive treatment was continued in 11 of 21 patients (unknown in one case). In patients with MTX monotherapy (n = 9), immunosuppressive treatment was interrupted in only 2 patients. If two immunosuppressive medications were used, immunosuppressive medication was interrupted in 7 of 11 patients. Two patients continued their baseline medication despite occurrence of complications (1 cellulitis, 1 cerebellitis). After interruption, the immunosuppressive therapy was restarted within one (n = 5), two (n = 4) or three weeks (n = 1) after VZV manifestation. Retrospective assessment of overall disease activity by the treating physician (expert opinion) six months after VZV manifestation was reported to be unchanged in 15 patients, less active in three patients, more active in three patients and unknown in one patient.

## Discussion

The aim of our study was to assess the presentation and complications of VZV manifestation in children with rheumatic autoimmune diseases under immunosuppression. The clinical course of varicella and herpes zoster and their potential complications is well known, but solid information about the clinical presentation and the efficacy of treatment in these patients is scarce. Switzerland is a country with a high incidence of varicella, because varicella vaccination is only recommended for the 11 to 39 years old healthy population with a negative varicella history or negative VZV-specific IgG antibodies [4]. Therefore, most of the children experience varicella with a peak of VZV seroconversion at 3 to 8 years [2, 5]. The majority of patients with pediatric rheumatic diseases, especially JIA, is also diagnosed within the same age range. As iatrogenic immunosuppression and immune dysfunction are risk factors for complications during VZV infection, the current Swiss Federal Office of Public Health vaccination plan recommends varicella vaccination for patients with autoimmune diseases without immunosuppressive treatment. However, these recommendations frequently interfere with an urgent need to start immunosuppressive medication, often leading to a postponement of vaccination and thereby increasing the risk for varicella.

Occurrence of varicella under iatrogenic immunosuppression in children with juvenile idiopathic arthritis has been reported in several studies addressing the safety of bDMARDs [3]. However, detailed published data addressing the outcome of such cases are scarce. Wiegering et al. reported two immunocompromised patients with JIA that showed varicella without complications under aciclovir treatment [6]. In a study addressing the safety of etanercept in 25 children with JIA under four years of age, two patients developed varicella, of whom one was complicated by necrotizing fasciitis [7]. In our study, the clinical presentation of varicella was typical or mild in most of the patients. However, four of the 16 patients with varicella developed complications which occurred under both single-drug and combination therapy. Cellulitis was the most frequent complication, as it is the case in the healthy population [1, 8, 9]. Systemic complications presenting as sepsis were observed in one patient, which is a rare complication of varicella in immunocompetent patients [10, 11]. Due to the retrospective nature of this study, a calculation of the complication rate was not possible. However, it may exceed the reported rate of severe complications of about 1/100′000 from population based European studies [12]. 

Interestingly, two patients with varicella had previously received a first dose of varicella vaccine two and four weeks before the introduction of methotrexate, respectively, and one patient 8 years of age had a history of previous varicella at 6 months of age. In large studies, a single dose of varicella vaccine regimen has been shown to be only 80 to 85 % effective in preventing varicella [13, 14], therefore, a two-dose schedule has been implemented in most of the countries, including Switzerland. Cell-mediated immunity plays a crucial role in controlling varicella disease and in inducing protection [15, 16], and the immunosuppressive treatment possibly influenced VZV immunity in these two patients [17].

Concerning antiviral treatment, only 12 of the 22 patients had been treated with aciclovir or valaciclovir. The absence of antiviral treatment in the remaining 10 patients did not result in any complication.

We were surprised that none of the 22 patients had been treated with anti-VZV immunoglobulins. Two possible explanations may contribute to this finding: First, awareness of this therapeutic approach was potentially not yet sufficiently established in the early study period [18, 19]. Second, the time window for a post-exposure treatment (usually within 96 h) might have been missed in some of these patients.

Herpes zoster in healthy children is rare. Recently, Weinmann et al. [20] estimated an incidence of HZ of 230 per 100′000 patient-years in a healthy population. In non-VZV vaccinated children with JIA exposed to biologics, a German register-based study reporting on 17 herpes zoster events calculated an incidence of 3.1/1000 patient years (95 % CI, 1.9–4.9) [21]. Two of the patients with a known outcome experienced complications (intercostal neuralgia and recurrent herpes zoster). The largest study reporting on JIA patients and herpes zoster has been published by Beukelman et al. [22]. Using US Medicaid administrative claims data on 8500 JIA patients with 360000 children diagnosed with attention deficit hyperactivity disorder used as comparator group, this study found an incidence rate of 225 per 100′000 person years and an incidence risk ratio of 2.1 (95 % CI, 1.4–3.0). Beukelman et al. did not observe a significant association between the occurrence of herpes zoster and the use of a specific immunosuppressive medication.

Our study reported HZ in six children without further complications. Of note, four presented early within less than 12 months after onset of the immunosuppressive therapy. This observation underscores that iatrogenic immunosuppression may favor reactivation of latent Herpesviridae diseases [23].

Data on hospitalization for varicella in childhood, including Switzerland, report rates of 9–10/1000 patients [24]. In our study, 27 % of the patients were admitted to hospital, either for a VZV-associated complication or an intravenous antiviral treatment. Thus, the hospitalization rate of immunosuppressed patients is influenced by the decision of an intravenous antiviral treatment and could be, as in this study, remarkably higher than in the general population.

Half of the pediatric rheumatologists did continue immunosuppressive medication during VZV manifestation. Most of the specialists continued methotrexate treatment, as this medication has a long half-life. Combination therapy was associated with a higher interruption of therapy; two thirds of the patients paused the immunosuppressive medication during VZV manifestation. The heterogeneous therapeutic approach probably reflects the absence of established guidelines in this clinical situation.

Our study has several limitations. First, even though collected from a register, our data are of retrospective nature, and not all data were available concerning the management of VZV manifestations. Second, the retrospective setting implies a risk of underreporting, and a calculation of VZV manifestation incidence und complication rates was not possible from the existing data. Third, assessment of overall disease activity of the underlying autoimmune disease subsequent to VZV disease was not possible to be based on validated disease activity scores. Thus, potential changes in disease activity represent only an expert opinion, reflecting the clinical follow up of an individual patient. Fourth, our study was too small to allow recommendations for VZV treatment and handling of immunosuppressive medication during VZV infection in immunosuppressed patients with rheumatic autoimmune diseases. This should be assessed in larger studies in the future.

## Conclusion

In conclusion, this study shows that the clinical presentation of VZV disease in children with rheumatic autoimmune diseases under immunosuppression may be altered in respect to complication rate and hospitalization rate. To prevent the occurrence of varicella or herpes zoster, thorough assessment of VZV infection or vaccination prior to immunosuppression and correct and timely VZV vaccination according to national guidelines is essential to minimise VZV complications in this vulnerable population.

## Abbreviations

bDMARDs: biological disease-modifying antirheumatic drugs
cDMARDs: conventional disease-modifying antirheumatic drugs
CI: confidence interval
MTX: methotrexate
VZV: varicella zoster virus
